# Personalized mechanical ventilation guided by lung ultrasound in patients with ARDS: a pilot phase of a randomized clinical trial

**DOI:** 10.1186/s40635-025-00835-8

**Published:** 2025-12-22

**Authors:** Jante S. Sinnige, Marry R. Smit, Mohammad J. Alam, Mohammed N. H. Chowdhury, Vasco Costa, Heloísa S. M. B. de Castro, Dominik Daszuta, Daan F. L. Filippini, Aniruddha Ghose, Harm-Jan de Grooth, Lars Hein, Greet Hermans, Thomas Hildebrandt, Theis Skovsgaard Itenov, Eleni Ischaki, Peter Klompmaker, John Laffey, Aisling McMahon, Bairbre McNicholas, Amne Mousa, Frederique Paulus, Ulf Gøttrup Pedersen, Mariangela Pellegrini, Marco Pezzuto, Pedro Póvoa, Charalampos Pierrakos, Luigi Pisani, Oriol Roca, Marcus J. Schultz, Savino Spadaro, Konstanty Szuldrzynski, Evangelia Theodorou, Pieter R. Tuinman, Christian A. Wamberg, Claudio Zimatore, Lieuwe D. J. Bos

**Affiliations:** 1 Department of Intensive Care, Amsterdam University Medical Centres (UMC), University of Amsterdam, Meibergdreef 9, 1105 AZ Amsterdam, The Netherlands; 2 Department of Critical Care Medicine & Emergency, Apollo Imperial Hospitals, Chattogram, Bangladesh; 3 Department of Intensive Care, Marine City Medical College Hospital, Chattogram, Bangladesh; 4 Department of Intensive Care, Hospital de São Francisco Xavier, CHLO, Lisbon, Portugal; 5 NOVA Medical School, CHRC, NOVA University of Lisbon, Lisbon, Portugal; 6 Department of Intensive Care, Centro Hospitalar Universitário de Santo António, Porto, Portugal; 7 Department of Anaesthesiology and Intensive Care, National Institute of Medicine of the Ministry of Interior and Administration, Warsaw, Poland; 8 Department of Medicine, Chattogram Medical Centre, Chattogram, Bangladesh; 9 Intensive Care Center, UMC Utrecht, Utrecht, The Netherlands; 10 Department of Anaesthesia and Intensive Care, Copenhagen University Hospital-North Zealand, Copenhagen, Denmark; 11 Medical Intensive Care Unit, University Hospitals Leuven, Louvain, Belgium; 12 Department of Cellular and Molecular Medicine, KU Leuven, Louvain, Belgium; 13 Department of Intensive Care, Zealand University Hospital, Roskilde, Denmark; 14 Department of Anesthesiology and Intensive Care, Copenhagen University Hospital - Bispebjerg and Frederiksberg, Copenhagen, Denmark; 15 Department of Clinical Medicine, University of Copenhagen, Copenhagen, Denmark; 16 First Department of Intensive Care Medicine, University of Athens Medical School, Athens, Greece; 17 Department of Intensive Care, Amsterdam UMC, Vrije Universiteit, Amsterdam, The Netherlands; 18 Amsterdam Cardiovascular Sciences, Amsterdam UMC, Vrije Universiteit Amsterdam, Amsterdam, The Netherlands; 19 Anaesthesia and Intensive Care Medicine, Galway University Hospitals and School of Medicine, University of Galway, Galway, Ireland; 20 Department of Critical Care Medicine, Mater Misericordiae University Hospital, Dublin, Ireland; 21 Department of Anaesthesia and Intensive Care Unit, Zealand University Hospital, Køge, Denmark; 22 Department of Surgical Sciences, Uppsala University, Uppsala, Sweden; 23 Intensive Care Unit, Akademiska Sjukhuset, Uppsala University Hospital, Uppsala, Sweden; 24 Department of Intensive Care, Ospedale Generale Regionale F. Miulli, Acquaviva Delle Fonti, Bari, Italy; 25 Center for Clinical Epidemiology and Research Unit of Clinical Epidemiology, OUH Odense University Hospital, Odense, Denmark; 26 Department of Intensive Care, Brugmann University Hospital, Université Libre de Bruxelles, Brussels, Belgium; 27 Department of Precision-Regenerative Medicine and Jonic Area (DiMePRe-J), Section of Anesthesiology and Intensive Care Medicine, University of Bari “Aldo Moro”, Bari, Italy; 28 Servei de Medicina Intensiva, Hospital Universitario de Bellvitge, L’Hospitalet de Llobregat, Barcelona, Spain; 29 Ciber Enfermedades Respiratorias, Ciberes, Insituto de Salud Carlos III, Madrid, Spain; 30 Mahidol Oxford Tropical Medicine Research Unit (MORU), Mahidol University, Bangkok, Thailand; 31 Nuffield Department of Medicine, University of Oxford, Oxford, UK; 32 Clinical Department of Cardiothoracic Vascular Surgery Anesthesia and Intensive Care Medicine, Medical University Wien, Vienna, Austria; 33 Anesthesiology and Intensive Care, Department of Translational Medicine, Faculty of Medicine and Surgery, University of Ferrara, Ferrara, Italy; 34 Department of Pulmonology, Amsterdam UMC, University of Amsterdam, Amsterdam, The Netherlands; 35 Laboratory of Experimental Intensive Care and Anaesthesiology (L.E.I.C.A.), University of Amsterdam, Amsterdam, The Netherlands

**Keywords:** Respiratory distress syndrome, Ultrasonography, Artificial respiration, Precision medicine

## Abstract

**Background:**

The “Personalized Mechanical Ventilation Guided by Lung UltraSound in Patients with Acute Respiratory Distress Syndrome” (PEGASUS) study aims to evaluate personalized mechanical ventilation (MV) in patients with acute respiratory distress syndrome (ARDS) compared to the standard of care. However, misclassification and misaligned MV strategies were shown to be harmful. We therefore aimed to assess the interobserver agreement of lung ultrasound (LUS) between the local investigator and an expert panel in classifying ARDS subphenotypes alongside protocol adherence and safety endpoints, as a pilot phase of the ongoing PEGASUS study.

**Methods:**

The first 80 mechanically ventilated patients with moderate-to-severe ARDS were enrolled in the ongoing PEGASUS study, a randomized clinical trial (RCT), and were included in the pilot phase. Focal or non-focal subphenotypes were classified using a LUS. Positive end-expiratory pressures (PEEP), tidal volumes (VT), the application of recruitment manoeuvres, and proning were performed according to randomization arm and subphenotype. Safety limits for MV followed current guidelines. Agreement in subphenotype classification between local investigators and a panel of three experts was evaluated using Cohen’s *κ* coefficient.

**Results:**

In 68 out of 80 exams, the images were of sufficient quality for assessment. The interobserver agreement for the lung morphology had a Cohen’s kappa of 0.72 (95% CI 0.53–0.9) and accuracy of 88% between local investigator and the expert panel. Misclassification occurred in 8/68 exams (11.8%). Among these 8 misclassified cases, 6 (75%) also showed disagreement between experts due to different LUS scores of the anterior regions. Tidal volume and PEEP were generally set according to the protocol. An exception was the TV in the non-focal ARDS patients randomized to personalized MV, where the median (6.2 ml/kg/PBW) was above the target range (4–6 ml/kg/PBW). Patients exceeding safety limits of MV were low.

**Conclusion:**

In the pilot phase of an ongoing international subphenotype-targeted RCT, we found that local investigators’ assessments agreed with expert panel consensus assessments in the large majority of cases, and nearly always when the expert panel assessment was unanimous. Protocol adherence was sufficient, but tidal volume in the non-focal subphenotype deserves attention during continuation of the study.

*Trial registration*: The study was registered on clinicaltrial.gov (ID: NCT05492344, date 2022-08-05).

**Supplementary Information:**

The online version contains supplementary material available at 10.1186/s40635-025-00835-8.

## Background

Acute respiratory distress syndrome (ARDS) is a common cause of hypoxemic respiratory failure with high morbidity and mortality on the intensive care unit (ICU), often requiring mechanical ventilation (MV) for patient support [1, 2]. The current guidelines for ventilation strategies in ARDS patients are mostly driven by the severity of hypoxemia based on PaO_2_/F_I_O_2_ and do not routinely rely on ARDS subphenotypes [3, 4]. However, identifying ARDS subphenotypes based on “focal” or “non-focal” lung morphology can potentially optimize MV strategies and outcomes for individual patients [5]. The PEGASUS study, “Personalized mechanical ventilation guided by lung ultrasound in patients with acute respiratory distress”, is an international randomized clinical trial (RCT) designed to assess the effect of personalized ventilation on all-cause mortality compared to the standard of care in patients with ARDS [6].

While the LIVE study demonstrated that personalized ventilation strategies can improve patients outcomes, it also revealed that misclassification of morphology subphenotypes and subsequent misaligned treatment could harm patients leading to worse outcomes [5]. The high rate of misclassified patients was primarily caused by the use of chest X-rays (CXR) to distinguish between “focal” (basolateral posterior consolidation) and “non-focal” (opacities scattered throughout the lung) ARDS subphenotypes, a method known for its poor diagnostic accuracy [7]. Computed tomography (CT) remains the gold standard for identifying radiologic subphenotypes. However, it requires transporting critically ill patients, is not available in all hospitals, and necessitates interpretation by an experienced physician [8, 9]. Lung ultrasound (LUS) has a high diagnostic accuracy (89%) compared to CT [10, 11] in classifying patients. In contrast to CXR, LUS is well-suited to evaluate the dorso-ventral gradients, and an algorithm based on ventral aeration pattern can differentiate between “focal” and “non-focal” lung morphology. PEGASUS is the first study to incorporate this LUS algorithm prospectively in a clinical setting with treatment implications.

To prevent misclassification driving negative study results, we considered it critical to incorporate a pilot phase within the PEGASUS study with the objective to evaluate the agreement of LUS between the local research teams and an expert panel. Furthermore, the pilot phase provided an opportunity to assess protocol adherence, ensuring consistent implementation across all participating sites. It also evaluated patient safety by identifying any risks or adverse outcomes associated with the study intervention.

We hypothesize that the interobserver agreement in LUS subphenotype classification is high, the protocol is well adhered to, and MV is delivered within established safety limits. Therefore, we compare the classification of local investigators to a central team of LUS experts. In addition, we evaluate adherence to and feasibility of the study protocol and quantify whether personalized MV can be delivered within the “safe limits” of ventilation defined by the protocol.

## Methods

This is a pilot phase compromising the first 80 of the total 538 patients that will be included in the ongoing PEGASUS study, a phase 3 international RCT. The patients are being recruited from twelve general or academic hospitals across eight countries. The study protocol was published [6] and registered on clinicaltrial.gov (ID: NCT05492344) prior to initiation of recruitment. The protocol was approved by the institutional ethics committee of the Amsterdam University Medical Center (ref: 2022.0148-NL79110.018.21) and by the ethics committees of participating centres. A data safety monitoring board (DSMB) was installed before recruitment.

### Participants

Patients could participate in the pilot phase of the PEGASUS study when they were mechanically ventilated and admitted to a participating ICU with moderate or severe ARDS according to the Berlin definition [12] using CT and CXR as radiology modality. All exclusion criteria can be found in the online supplement on page 5. Deferred or informed consent was obtained for all the included subjects from a legal representative of the patient. Patients were randomly assigned to either the personalized ventilation or control arm in a 1:1 ratio using Castor Electronic Data Capture (EDC) (https://www.castoredc.com), with stratification applied only by centre.

### Lung ultrasound

**Fig. 1:**
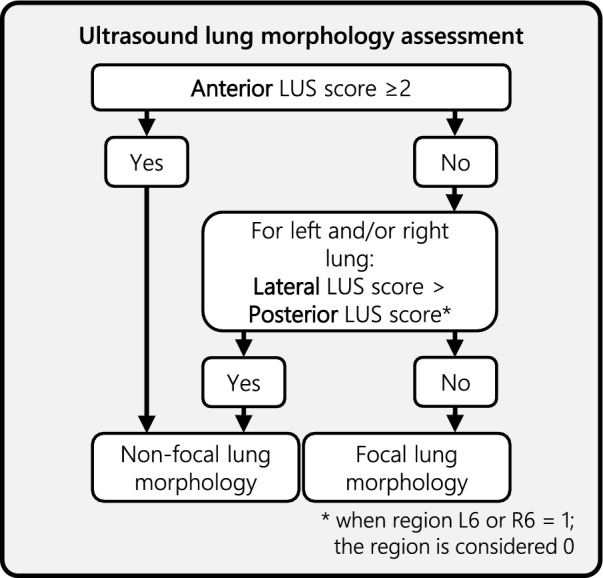
Ultrasound lung morphology assessment. This algorithm is used to assess lung morphology through ultrasound in eligible patients. The model is based on a 12-region LUS exam, which includes two anterior, two lateral, and two posterior fields on both sides of the thorax. Each region can be scored according to the following patterns: A-pattern (horizontal lines, scored as “0”), B1-pattern (more than two vertical lines extending from the pleura, covering less than 50% of the pleural line, scored as “1”), B2-pattern (multiple vertical lines covering more than 50% of the pleural line, scored as “2”), or C-pattern (consolidation greater than 2 cm, scored as “3”). In the caudal posterior regions (L6 and R6), a B1-pattern is scored as an A-pattern due to the frequent non-pathological appearance of B1-patterns in ventilated, bedbound ICU patients. LUS = lung ultrasound

A 12-region LUS exam (six regions per hemithorax) was performed using a standardized protocol in the supine position to determine lung morphology (online data supplement on page 6). To reduce the risk of subphenotype misclassification, the positive end-expiratory pressure (PEEP) was set at 5 cm H₂O. A maximum of 8 cm H₂O PEEP was allowed if 5 cm H₂O was deemed clinically unfeasible. Each lung region was scored as: an A-pattern (horizontal lines, scored as “0”), a B1-pattern (more than two vertical lines extending from the pleura but covering less than 50% of the pleural line, scored as “1”), a B2-pattern (multiple vertical lines covering more than 50% of the pleural line, scored as “2”), or a C-pattern (consolidation greater than 2 cm, scored as “3”). “Focal” or “non-focal” lung morphology was assessed using the algorithm in Fig. 1. Participating centres received online training through an e-learning program followed by an exam, case-based assessments, and support for the first two patients. The initial scores, before feedback from the steering committee, were used as initial assessment to evaluate of the interobserver agreement. The LUS video clips of all patients were saved and stored for the second assessment. This was performed by an expert panel of three assessors, who all evaluated the images individually while remaining blinded to the result of the initial assessment. In the case of disagreement among the three experts, the images were discussed between experts to reach consensus.

### Ventilation strategy

A synopsis of the PEGASUS MV protocol, based on the randomization arm and lung morphology, is presented in Table 1. A detailed description of the ventilation strategy can be found in the published protocol [6]. Good protocol adherence was defined when the ventilator was set according to the protocol. A “Safety protocol” was used when a patient could not be ventilated according to the protocol because safe limits of MV were not guaranteed after optimizing the ventilator (e.g., plateau pressure > 30 cm H₂O, tidal volume > 10 mL/kg predicted body weight (PBW), or pH < 7.25), or when patients experienced severe hypoxemia requiring rescue therapies. Patients were released from the protocol when they were expected to be extubated within 48 h based on ventilation parameters or when patients were placed on extracorporeal membrane oxygenation.

**Table 1:** Ventilation strategies based on our study protocol, stratified by randomization arm and lung morphology

	Standard of care	Personalized group	
		“Focal”	“Non-focal”
Mode of ventilation	Pressure controlled, volume controlled or pressure support	Pressure controlled, volume controlled or pressure support	Pressure controlled, volume controlled or pressure support
Tidal volume	6 mL/kg PBW	6–8 mL/kg PBW	4–6 mL/kg PBW
PEEP	Low PEEP/high F_I_O_2_ [13]	≤ 9 cm H_2_O	≥ 15 cm H_2_O
Recruitment manoeuvre	Only for rescue	Only for rescue	Daily
Prone positioning	PaO_2_/F_I_O_2_ < 150	PaO_2_/F_I_O_2_ < 200	PaO_2_/F_I_O_2_ < 150

Formula for calculating the tidal volume size with PBW are 50 + 0.91 × (centimetres of height − 152.4) for males and 45.5 + 0.91 × (centimetres of height − 152.4) for females

PBW, predicted body weight, PEEP, positive end-expiratory pressure, PaO_2_, partial pressure of oxygen in arterial blood, F_I_O_2,_ fraction of inspired oxygen

### Study endpoints

The primary endpoint of the pilot phase was the interobserver agreement between the treating physician and an expert panel in the assessment of lung morphology using LUS exams. The secondary endpoint of this pilot phase was protocol adherence according to the lung morphology and randomization arms (standard of care or personalized strategy). Protocol acceptability was defined by the willingness of clinicians to randomize patients, and the willingness of patients’ families to give consent. Furthermore, we assessed the clinical feasibility of the standardized LUS exam in a multicentre setting by the time required to perform the LUS exam, the practicality of LUS in the proposed setting, and the percentage of correctly performed LUS exams. Lasty, we assessed the percentages of available data, and occurrence of preset safety endpoints defined as the prevalence of plateau pressure > 30 cmH_2_O, tidal volumes > 10 mL/kg PBW in controlled ventilation mode, ventilator-associated pneumonia (VAP), and pneumothorax (online data supplement on page 28).

### Statistical analysis

Prior to initiation, we determined that a minimum of 20 patients in each personalized treatment group would be required to evaluate clinical feasibility and adherence to the study protocol. Given the anticipated 1:1 ratio between ‘focal’ and ‘non-focal’ patients, as well as between the intervention and control arm, a total sample size of 80 patients was necessary for this pilot phase. Cohen’s Kappa (*κ*) coefficient was used to assess interobserver agreement between the treating clinicians and the expert panel. Agreement was considered poor (< 0), slight (0.01–0.20), fair (0.21–0.40), moderate (0.41–0.60), substantial (0.61–0.80) or almost perfect (0.81–1.00). We anticipated an interobserver agreement among experts with a *κ* value of 0.9. To detect a relevant reduction in *κ* to 0.7 between the expert panel and bedside clinicians, a total of 60 patients were required to achieve 80% power at a one-sided α level of 0.05. Variables are expressed as frequencies and percentages, means and standard deviations or medians and inter quartile ranges (IQR) whenever appropriate. The analyses were performed using R (version 4.3.2,www.r-project.org). Changes in the statistical analysis compared to our published protocol are described in online data supplement on page 29.

## Results

**Fig. 2:**
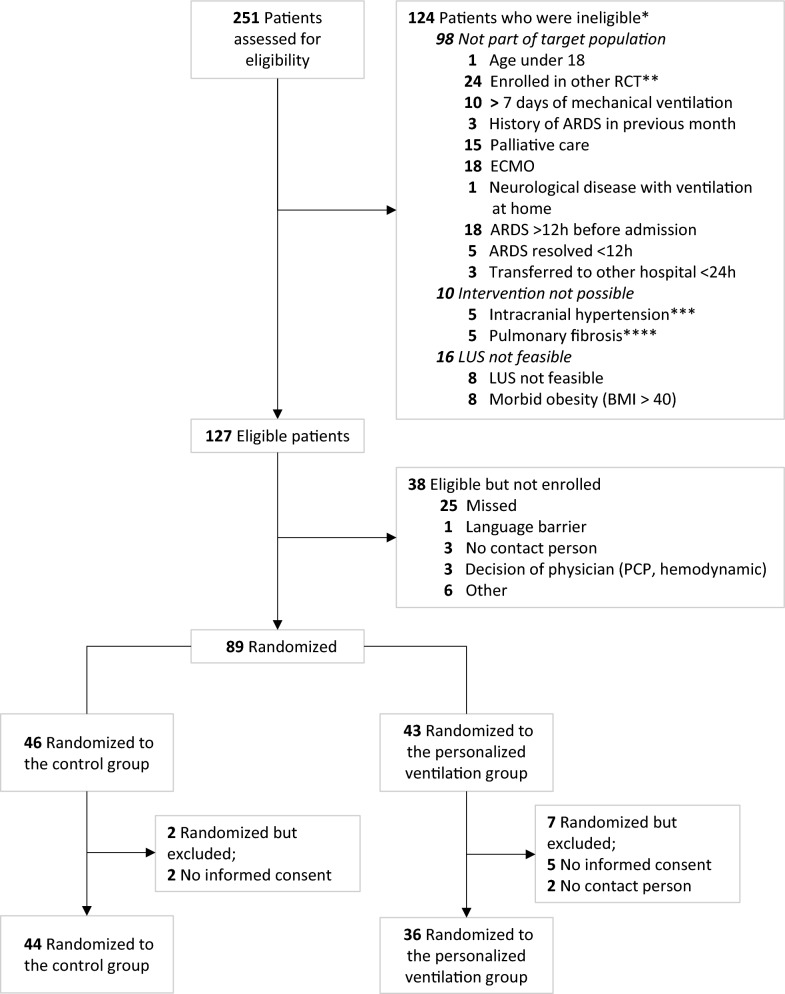
Flow of participants in the pilot phase of the PEGASUS study. RCT, randomized clinical trial; LUS, lung ultrasound; ARDS, acute respiratory distress syndrome; BMI, body mass index; ECMO, extracorporeal membrane oxygenation. *If multiple exclusion criteria were present, the main reason of exclusion was reported. **Patient could not participate if they were enrolled in a RCT with similar endpoints. ***Confirmed by an Intracranial Pressure device. ****With a vital capacity < 50% (severe or very severe)

The first 80 patients enrolled in the PEGASUS study were recruited between August 9th, 2022, and April 23rd, 2024. During this period, 251 patients met the inclusion criteria, of whom 124 (49.4%) also met at least one exclusion criteria (Fig. 2). From the excluded patients, 16 (6.4%) were not included because LUS was not feasible (e.g., subcutaneous emphysema or morbid obesity). 38 patients were eligible but not enrolled resulting in 89 randomized patients (Fig. 2). The clinician decided against inclusion in three patients due to haemodynamic instability or concerns related to pneumocystis pneumonia and potential air trapping. Finally, seven of the 89 randomized patients (7.9%) were excluded because their families did not provide consent. Baseline characteristics are presented in Table 2, stratified for randomization arm. The most common cause of ARDS was pneumonia.

**Table 2:** Baseline characteristics of patients at inclusion

	Control group *n* = 44	Personalized ventilation group *n* = 36
Baseline characteristics		
Age (years)	62 (46–75)	60 (49–72)
Female (%)	20 (45%)	13 (36%)
Duration of ARDS before randomization (hours)	4 (2–10)	4 (3–8)
Duration of ventilation before randomization (days)	1 (1–2)	1 (0–1)
Clinical frailty score	3 (2–4)	2 (2–5)
SOFA score	8 (7–11)	9 (8–11)
Cause of ARDS		
Pneumonia	32 (73%)	28 (78%)
Non pulmonary sepsis	5 (11%)	4 (11%)
Aspiration of gastric contents	5 (11%)	8 (22%)
Major trauma	0 (0%)	0 (0%)
Pulmonary contusion	0 (0%)	1 (3%)
Pancreatitis	0 (0%)	1 (3%)
Inhalation injury	1 (2%)	0 (0%)
Severe burns	0 (0%)	0 (0%)
Non cardiogenic shock	0 (0%)	0 (0%)
Drug overdose	0 (0%)	1 (3%)
TRALI	2 (5%)	0 (0%)
Pulmonary vasculitis	0 (0%)	0 (0%)
Drowning	2 (5%)	0 (0%)
Ventilation data during LUS exam		
Supportive ventilation	10 (23%)	12 (33%)
PEEP (cmH_2_O)	8 (5–12)	5 (5–8)
Tidal volume (mL/kg (PBW))	6.6 (6–7.7)	6.4 (5.6–7.5)
PaO_2_/F_I_O_2_ ratio (mmHg)	118 (77–153)	115 (96–153)
Driving pressure (cmH_2_O)	15 (11–19)	12 (10–18)
Global LUS score	16 (12–21)	18 (13–22)
Focal ARDS	12 (27%)	11 (31%)

The table is stratified by randomization arm. Results are presented in median with IQR or number with percentages

ARDS, acute respiratory distress syndrome; SOFA, Sequential Organ Failure Assessment; TRALI, transfusion related acute lung injury; LUS, lung ultrasound; PEEP, positive end-expiratory pressure; PBW, predicted body weight; PaO2, partial pressure of oxygen in arterial blood; F_I_O_2_, fraction of inspired oxygen

Of the 80 LUS exams performed, the images of five exams were missing, and six exams were scored as insufficient quality to be included in the second assessment. The causes of inadequate quality included the use of only still images, short video clip durations, insufficient depth in the video clips, or more than 50% rib shadowing. Agreement between experts on lung morphology was observed in 60 cases after the first evaluation, while they disagreed about nine cases (13%). Of these, one exam was excluded because of poor image quality. The expert panel meeting reached consensus for the remaining eight cases. As a result, 68 (91%) of the 75 available exams met the quality criteria and were used to compare the score assigned by the local investigator and by the central expert team.

### Lung ultrasound agreement

Interobserver agreement for LUS between local investigator and expert panel consensus was substantial (Cohen’s *κ*: 0.72 (95% CI 0.53–0.90)), resulting in an accuracy of 88% (Table 3). Interobserver agreement between each expert and the conclusion of the expert panel was very good, with Cohen’s *κ* between 0.87 and 0.90. Misclassification occurred in eight out of 68 exams (11.8%). Among the eight misclassified patients, there was also disagreement between the three experts in six cases (75%). Misclassifications were more common when the local investigator identified minor abnormalities in the anterior field, resulting in a ventral lung aeration score at the border between focal and non-focal classification (Figure E1). In the two other patients, the expert panel was in complete agreement on the ARDS phenotype, which differed from the classification made by the local investigator. In one of these cases, the misclassification was identified shortly after randomization, and the treatment was adjusted accordingly. This leaves one out of 68 patients (1.5%) in whom there was a clear disagreement between local investigator and expert panel resulting in misaligned treatment. The LUS protocol was completed in a median time of 14.5 min [IQR 9, 19], with a median PEEP of 6 cm H₂O [IQR: 5, 10] during the exam. Twenty-one exams involved a PEEP greater than 8 cm H₂O. However, higher PEEP was permitted only for non-focal patients, as lowering the PEEP would not change the subphenotype.

**Table 3:**
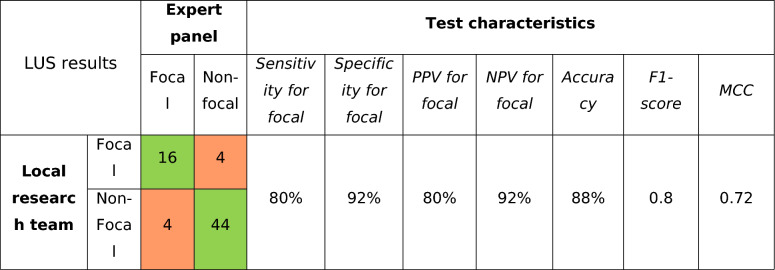
Distribution of patients based on their lung morphology classified by the local research team and expert panel

The green shading in the cross-tabulation indicates correctly classified patients, while the red shading represents incorrectly classified patients

LUS, lung ultrasound; PPV, positive predictive value; NPV, negative predictive value; MCC, Matthews correlation coefficient

### Compliance with the ventilation protocol

**Fig. 3:**
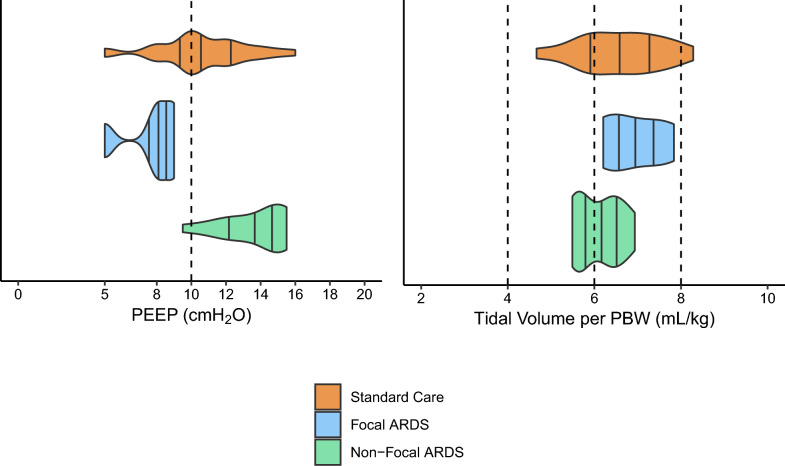
Median PEEP (cmH_2_O) and tidal volume per PBW (mL/kg) stratified for lung morphology and randomization arm. Ventilation data were included in this plot when patients were ventilated with a controlled mode, not on ECMO and not expected to be extubated. The dotted lines separate different ventilations strategies. ARDS, acute respiratory distress syndrome; PEEP, positive end-expiratory pressure; PBW, predicted body weight

Figure E2 displays protocol adherence for each patient, with each row representing an individual patient and showing their adherence to the protocol during the first seven days after randomization. Median duration of prone positioning was 15 h [IQR 9, 18] per session, and median number of recruitment manoeuvres performed per day was 1.5 [IQR 1, 3]. Figure 3 displays the median PEEP (cmH₂O) and tidal volume per PBW (mL/kg) for patients ventilated in controlled mode, excluding those on ECMO or expected to be extubated, over the first seven days after randomization. Most ventilation strategies were in line with the study protocol except for the tidal volume in the non-focal ARDS patients randomized for the personalized ventilation with a median of 6.2 [IQR 5.7–6.5] mL/kg PBW, while 4–6 mL/kg PBW was targeted. Additionally, the PEEP level in the same intervention arm appeared to be low (Fig. 3). However, physicians were allowed to decrease PEEP if oxygenation improved. Detailed ventilation parameters by day, stratified by randomization arm, subphenotype, and ventilation mode, are presented in Table E1.

### Complications and safety

During ICU stay, VAP developed in 18 patients (23%). Pneumothorax was diagnosed in six patients (8%). Among the 189 tidal volume measurements, one (0.5%) exceeded 10 mL/kg of PBW. Of the 132 plateau pressure measurements, three (2.3%) exceeded a plateau pressure of 30 cm H₂O. For the primary and secondary endpoints, missing values were all under 5%.

## Discussion

In this pilot phase of an international ongoing subphenotype-targeted RCT, our major finding was that there is sufficient agreement in the assessment of lung morphology in ARDS patients between the treating physicians and the expert panel, with an accuracy of 88%. About 10% of cases were misclassification, potentially caused by an intermediate subphenotype. The ventilation protocol adherence was sufficient, with median tidal volumes and PEEP closely aligned with the ventilation strategy. An exception was observed in the tidal volumes in the non-focal ARDS patients randomized to intervention arm. The prevalence of ventilator-specific complications (e.g., VAP and pneumothorax) was in line with the current literature [14, 15]. The percentage of ventilation parameters outside the “safe limits” of ventilation was low.

LUS has been extensively used to diagnose various pulmonary conditions [16]. However, the use of LUS in the classification of ARDS subphenotypes for personalized treatment is novel. We present the first study that prospectively incorporates such algorithm-based classification in an ongoing international RCT. Personalized ventilation based on ARDS subphenotypes has been investigated in the LIVE trial, but 20% of patients were misclassified [5]. The primary cause of misclassification in the LIVE trial was the reliance on CXR, which, even when interpreted by experts, is known to have limited diagnostic accuracy [7]. Therefore, the actual misclassification rate in that trial was likely higher as experts could not rely on the gold standard for ARDS subphenotype classification. In most cases where there was no agreement in the PEGASUS pilot phase presented here, there was also no consensus between the experts on morphology subphenotype allocation. This could suggest that these patients had an intermediate subphenotype. One could speculate that misaligned treatment in patients with intermediate subphenotypes is of less consequence.

In this study, the observed interobserver agreement is higher than the interobserver agreement that we presumed to be a clinically relevant decrease compared to experts. However, the confidence intervals of the observed kappa value include both the presumed kappa between experts and the presumed clinically relevant decrease in kappa, indicating uncertainty. When comparing our results to previous studies, it is important to emphasize that our findings were obtained in real-world clinical settings. In more detail, the bedside images were captured by local investigators with varying levels of experience, unlike previous studies that often involve LUS experts. Additionally, local investigators used bedside images for the assessment, while the expert panel reviewed only saved images, differing from studies using the same clips for both. While these factors may contribute to lower agreement in our study, they likely are not the core contributor. All participating physicians were trained through online training, and their knowledge of LUS interpretation was tested before starting patient recruitment [6]. Previous studies have shown that such training provides a good basis for scoring LUS images [17, 18]. Lower agreement is likely due to the difficulty to directly compare our results with previous studies, as most have focused on the interobserver agreement of aggregated scores, such as the LUS aeration score [9, 19–21]. Unlike these aggregated scores, our lung morphology classification is sensitive to minor changes in the ventral fields, which can alter the classification. In this light, the observed accuracy seems reasonable and reflects the clinical reality when a subphenotype-targeted MV strategy would be implemented broadly.

To determine the effect of personalized ventilation on outcomes in ventilated ARDS patients, it is essential to ensure good protocol adherence in the delivered ventilation strategy. The results in this pilot phase show visible differences in the ventilation strategies with regard to PEEP, tidal volume, prone positioning, and recruitment manoeuvres between “focal” and “non-focal” patients in the personalized ventilation arm, while the control arm is overlapping both subphenotypes. For the yng PEGASUS study, we will continue to optimize protocol adherence by providing centre-specific ventilation reports, ensuring that all sites are adequately trained and familiar with the protocol during the trial. Most ARDS studies rely on intention-to-treat analyses on the delivered ventilation strategy [13, 22, 23] and often do not report protocol adherence clearly. We plan to provide more details on protocol adherence in the final publication, which will be outlined in the statistical analysis plan to be published prior to study completion.

This study’s strengths include its prospective, randomized, multicentre recruitment across eight countries, which enhances the generalizability of the findings. The pilot phase was integrated into the main study protocol and published prior to the completion of patient inclusion, ensuring transparency. However, there are several limitations to consider. An important limitation is the unavailability of images from five LUS exams, four of which were from the initiating hospital due to an ultrasound machine malfunction during the pilot phase. Since the study team was well trained, we do not expect that these missing exams would have influenced the results. Additionally, seven exams had poor image quality during the second assessment. Most poor-quality images were still images received from sites with extensive experience in LUS. Therefore, this issue reflects a technical limitation rather than a problem with correct classification. When poor-quality images were received from less experienced sites, correct classification was ensured by conducting videocalls during a LUS exams with the local investigators and the steering committee. Case-based feedback was provided to improve image quality resulting in improvement with subsequent inclusions. A second limitation is the potential for post-randomization bias due to the deferred consent method. Although there were more objections to participation in the intervention arm, this was primarily due to the emotional distress of family members of critically ill patients, and patient representatives never mentioned the randomization arm. Therefore, we expect these differences to diminish as the trial progresses. Furthermore, the percentage of no consent compared to randomized patients is much lower in comparison to similar MV studies [24, 25]. Taken together, we think the presented results suggest good generalizability and limited evidence for bias.

The intriguing results of the LIVE trial clarified that misclassification is potentially dangerous in subphenotype-targeted intervention studies. The presented study has provided sufficient confidence to the study team to continue with the PEGASUS study without modifications to the main protocol. Regular assessments of protocol adherence and close monitoring of the first LUS exams during the initial phase of new centres will be crucial. A key insight from this pilot phase is the identification of a potential intermediate radiological ARDS subphenotype, for which the current two-way strategy of our LUS algorithm in dividing lung morphology may be inadequate. However, this is an uncommon phenomenon occurring in approximately 10% of patients, and such intermediate groups will always exist when subphenotypes are created [26]. We plan to investigate this further through a sub-study within the PEGASUS study, utilizing quantitative CT scan analysis with a post hoc evaluation on lung mechanics. Finally, this study demonstrates that delivering interventions based on lung ultrasound taught through remote training is feasible. This approach is particularly valuable in settings with limited access to CT imaging.

## Conclusion

In conclusion, we observed sufficient agreement between the local research teams and the expert panel in assessing lung morphology. An intermediate subphenotype was likely present in a minority of the patients. Protocol adherence was generally satisfactory; however, the deviation from the targeted tidal volume in the non-focal ARDS subphenotype in the intervention arm requires closer monitoring as the study progresses. There were no concerns regarding the prevalence of the predefined endpoints for safety.

## Supplementary Information

Supplementary Material 1. Description of data: This word document contains additional files showing the list van PEGASUS investigators, exclusion criteria, our standard operating procedure for lung ultrasound, definitions of complications or events, changes in statistical analysis, Figure E1—Total anterior LUS score in aligned and misaligned patients, Figure E2—Protocol adherence per patient, Table E1—Ventilation parameters per day, stratified for randomization arm, phenotype and mode of ventilation, Table E2—Cohen’s kappa between expert opinion and local research team vs. expert panel, and Table E3—Baseline characteristics of patients at inclusion stratified by subphenotype.

## Data Availability

The datasets used and/or analysed during the current study are available from the corresponding author on reasonable request.

## Abbreviations

ARDS: Acute respiratory distress syndrome
LUS: Lung ultrasound
MV: Mechanical ventilation
PBW: Predicted body weight
ICU: Intensive Care Unit
RCT: Randomized controlled trial
CT: Computed tomography
CXR: Chest X-rays
ECMO: Extra corporeal membrane oxygenation
PEEP: Positive end-expiratory pressure
VAP: Ventilator-associated pneumonia
IQR: Inter-quartile range
PaO_2_: Partial pressure of oxygen in arterial blood
FiO_2_: Fraction of inspired oxygen
SOFA: Sequential Organ Failure Assessment
TRALI: Transfusion related acute lung injury
PPV: Positive predictive value
NPV: Negative predictive value
MCC: Matthews correlation coefficient
BMI: Body mass index
