# Supplementary material for: Personalized mechanical ventilation guided by lung ultrasound in patients with ARDS: a pilot phase of a randomized clinical trial
Source: Intensive Care Med Exp. 2025 Dec 22;13:135. doi: 10.1186/s40635-025-00835-8 (PMC12719360; doi:10.1186/s40635-025-00835-8)
Supplement: Supplementary file 1 — Supplementary Material 1. Description of data: This word document contains additional files showing the list van PEGASUS investigators, exclusion criteria, our standard operating procedure for lung ultrasound, definitions of complications or events, changes in statistical analysis, Figure E1—Total anterior LUS score in aligned and misaligned patients, Figure E2—Protocol adherence per patient, Table E1—Ventilation parameters per day, stratified for randomization arm, phenotype and mode of ventilation, Table E2—Cohen’s kappa between expert opinion and local research team vs. expert panel, and Table E3—Baseline characteristics of patients at inclusion stratified by subphenotype. [file 40635_2025_835_MOESM1_ESM.docx]

Personalized mechanical ventilation guided by lung ultrasound in patients with acute respiratory distress syndrome; a pilot phase of a randomized clinical trial.

Jante S. Sinnige, Marry R. Smit, Mohammad J. Alam, Mohammed N. H. Chowdhury, Vasco Costa, Heloísa S. M. B. de Castro, Dominik Daszuta, Daan F. L. Filippini, Aniruddha Ghose, Harm-Jan de Grooth, Lars Hein, Greet Hermans, Thomas Hildebrandt, Theis Skovsgaard Itenov, Eleni Ischaki, Peter Klompmaker, John Laffey, Aisling McMahon, Bairbre McNicholas, Amne Mousa, Frederique Paulus, Ulf Gøttrup Pedersen, Mariangela Pellegrini, Marco Pezzuto, Pedro Póvoa, Charalampos Pierrakos, Luigi Pisani, Oriol Roca, Marcus J. Schultz, Savino Spadaro, Konstanty Szuldrzynski, Evangelia Theodorou, Pieter R. Tuinman, Christian A. Wamberg, Claudio Zimatore, and Lieuwe D. J. Bos for the PEGASUS investigators*.

*Online Data Supplement*

**Content**

[List of the PEGASUS investigators 3](#_Toc211615476)

[Exclusion criteria 5](#_Toc211615477)

[Standard operating procedure: Lung Ultrasound 6](#_Toc211615478)

[Changes in statistical analysis compared to our previously published protocol. 9](#_Toc211615479)

[Figure E1 – Total anterior LUS score in aligned and misaligned patients 10](#_Toc211615480)

[Figure E2 – Protocol adherence per patient. 11](#_Toc211615481)

[Table E1 – Ventilation parameters per day, stratified for randomization arm, phenotype and mode of ventilation. 13](#_Toc211615482)

[Table E2 - Cohen’s kappa between expert opinion and local research team vs. expert panel (as golden standard). 15](#_Toc211615483)

[Table E3. Baseline characteristics of patients at inclusion stratified by subphenotype 16](#_Toc211615484)

List of the PEGASUS investigators

| Institute | First Name | Surname |
| --- | --- | --- |
| Hospital de São Francisco Xavier, CHLO | David | Nora |
| Centro Hospitalar Universitário do Porto | Cristina | Torrão |
|  | Irene | Aragao |
| CHU Brugmann | Mircea Tamas | Talpos |
|  | Rachid | Attou |
| UZ Leuven | Shannon | Nicolai |
|  | Helga | Ceunen |
|  | Anneleen | Gerits |
|  | Marijke | Peetermans |
|  | Stefanie | Sente |
| University of Bari Aldo Moro | Salvatore | Grasso |
|  | Leonarda | Maurmo |
| University of Ferrara | Valentina | Alvisi |
| Regional General Hospital F. Miulli | Adalgisa | Caracciolo |
|  | Fabrizia | Massaro |
|  | Giovanna | Magnesa |
| Centralny Szpital Kliniczny MSWiA | Miłosz | Jankowski |
|  | Joanna | Janowska |
|  | Konrad | Zuzda |
| Nordsjællands Hospital | Morten | Bestle |
|  | Lars | Christensen |
|  | Mikkel | Allingstrup |
|  | Sanne | Lauritzen |
| Bispebjerg Hospital | Anna Marie | Friis Pedersen |
|  | Diana | Bertelsen |
| Sjællands University Hospital | Lone Museus | Poulsen |
|  | Louise Stenbryggen | Herløv |
|  | Jacob Vad | Jensen |
|  | Lisbeth | Christiansen |
| Park Taulí University Hospital | Marina | Garcia de Acilu |
| Hospital Universitari de Bellvitge | Marta | Huguet |
| Hospital Clínic de Barcelona | Antoni | Torres |
|  | Enric | Barbeta |
| University of Galway | Camilla | Giacomini |
|  | Celia | Thomas |
|  | Martina | Carey |
|  | Peter | Moran |
|  | Eoin | Young |
|  | John | Bates |
|  | Lauren | Ferguson |
|  | Reginald | Caldicott |
|  | Deepika Rani | Basappakokati |
| Mater Misericordiae University Hospital | Paul | Bergin |
| The Adelaide and Meath Hospital | Yvelynne | Kelly |
|  | Sabina | Mason |
| Akademiska Uppsala University Hospital | Kristin Jona | Bjarnadottir |
|  | Rafael | Kawati |
| Evaggelismos Hospital | Harry | Giannopoulos |
|  | Angeliki | Kanavou |
|  | Ilias | Siempos |
| University Hospital of Heraklion | Katerina | Vaporidi |
| Marine City Medical College Hospital | Nahid | Hassan |
|  | Nahid | Nowroz |
|  | Samarjit | Barua |
| Imperial Hospital | Arif | Uddin Ahmed |

Exclusion criteria

- Diagnosis of ARDS for more than 12 hours
- ARDS resolved within 12 hours before inclusion
- Participating in other interventional studies with conflicting endpoints
- Aged below 18
- Conditions in which LUS is not feasible or possible (e.g. subcutaneous emphysema, thoracic wounds, prone positioning)
- Mechanically ventilated for longer than seven consecutive days in the past 30 days
- History of ARDS in the previous month
- A body-mass index higher than 40 kg/m²
- Intracranial hypertension
- A broncho-pleural fistula
- Chronic respiratory diseases requiring long-term oxygen therapy or respiratory support
- Pulmonary fibrosis with a vital capacity < 50% (severe or very severe)
- Receiving palliative care
- Receiving or planned to receive veno–venous, veno–arterial or arterio–venous extracorporeal membrane oxygenation
- Received invasive ventilation in home setting due to a neurological disease
- Previously randomized in this study.

Standard operating procedure: Lung Ultrasound

**Classification of Lung Morphology**

1. Start the lung ultrasound (LUS) machine.
2. Choose the linear probe, use of other probes is allowed when use of the linear probe does not result in assessable LUS images.
3. Choose the lung setting on the ultrasound machine. Most ultrasound machines have software that will minimize artifacts. For the assessment of the lungs, we would like to preserve these artifacts.
4. Place the patient in a semi-recumbent position (figure 1) and place the arms of the patient. If the patient was just placed from a prone to a supine position, please wait 30 minutes before the start of the LUS exam to mobilize fluid that has moved to the anterior lung fields due to the prone positioning towards the posterior lung fields. Position the patient's arms as shown in figure 2. This makes performing the ultrasound easier.

| 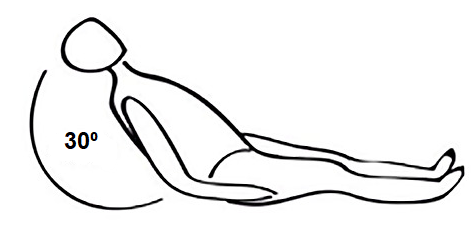 |
| --- |
| **Figure 1. Semi-recumbent position.** |

| 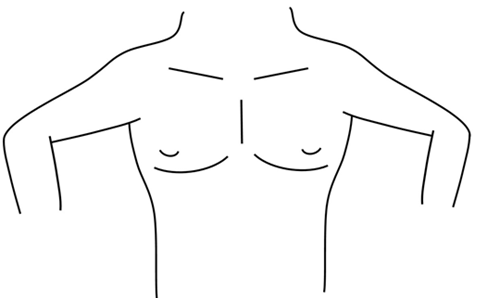 |
| --- |
| **Figure 2. Placing of the arms of the patient.** |

1. After pre-oxygenation and increasing the FiO_2_, set the PEEP to 5 cm H_2_O. If too much desaturation is expected with a PEEP of 5 cm H_2_O, the LUS exam can also be performed at a maximum PEEP level of 8 cm H_2_O.
2. Start the LUS exam by placing the probe longitudinal on region of interest with the pointer facing towards the cranial side of the patient.
3. Identify the pleural line between the ribs.
4. Set the depth at 10 cm, then adjust it in order to have the pleural line at 1/3 of the screen.
5. Focus should be on pleural line or on the point of interest if any alteration (i.e. consolidation).
6. Gain adjusted such that the pleural line is clearly visible but not overexposed.
7. Turn the probe with 90 degrees in which the pointer will face te right side of the patient.
8. Asses the lung aeration score of the region using the score below (figure 3).

| 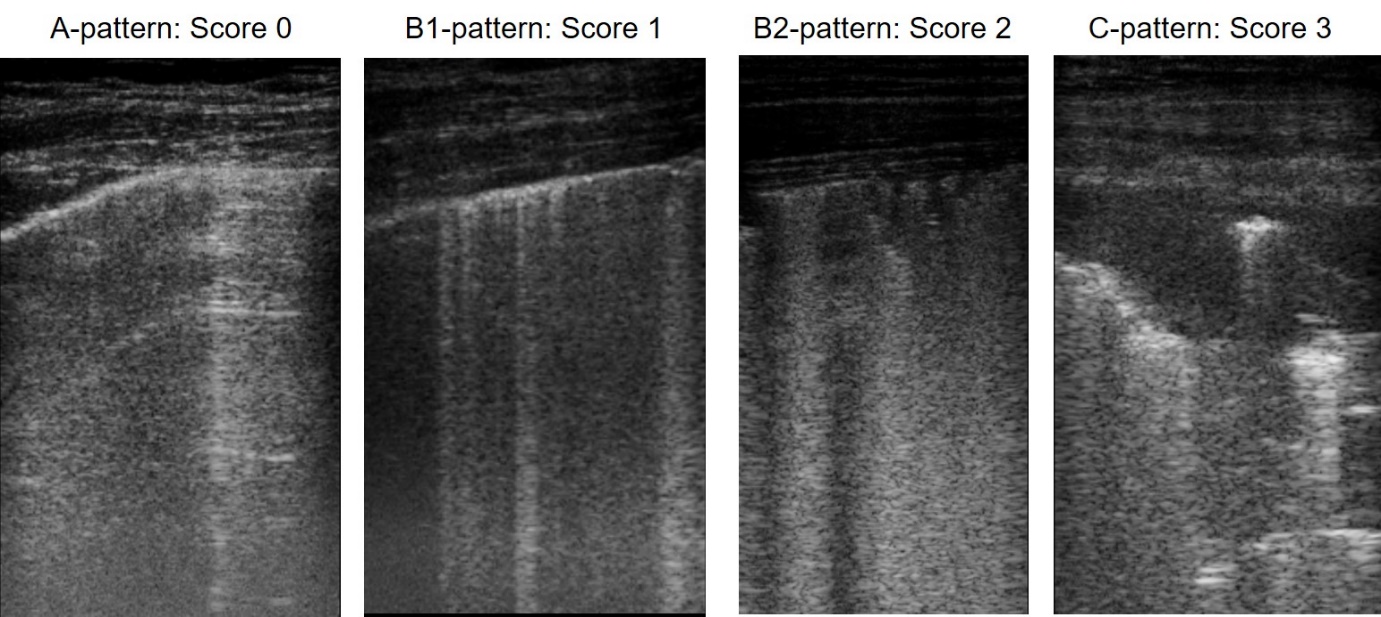 |
| --- |
| ***Figure 3****. Score 0: “A-pattern” (i.e., repeating horizontal A-lines parallel to the pleural line, suggesting normal aeration). Score 1: a “B-pattern” (i.e., three or more vertical B-lines starting from the pleural line and reaching the bottom of the screen, suggesting partial loss of aeration) and B-lines are well-spaced and cover ≤ 50% of the pleural line, Score 2: if B-lines cover ≥ 50% of the pleural line. Score 3: a “C-pattern” when the consolidation is > 2cm (i.e., consolidation, suggesting near-complete to complete loss of aeration).* |

1. Save the image as a clip when the image is of sufficient quality to score lung aeration.
2. Repeat this for the twelve different regions of the lungs, six locations for each hemithorax, shown on the picture below (figure 4).

| 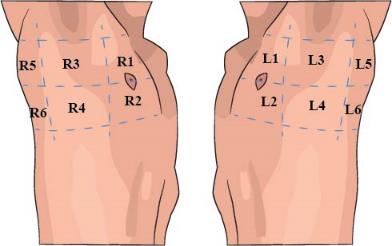 |
| --- |
| **Figure 4 – Twelve regions to perform the LUS.** |

1. When all regions have been scored, the results can be entered into the Castor database. This will automatically assess the lung morphology phenotype by using the flow diagram below (figure 5).

| 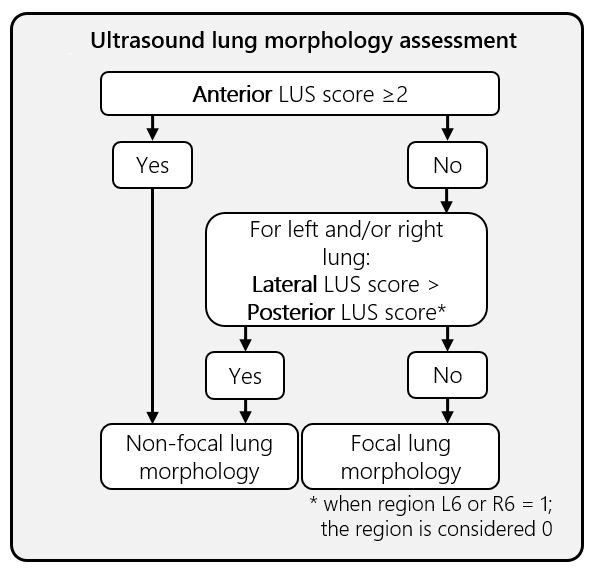 |
| --- |
| **Figure 5 – Assessment of lung morphology**. |

1. If due to any reason a region could not have been scored, the lung morphology phenotype of the patient must be assessed by the investigator him-/herself. The missing score can be complemented by using the scores of the other fields in the same area (anterior 1&2, lateral 3&4 or posterior 5&6). The missing region needs to be complemented for the morphology classification, but in the Castor database the region should be registered as missing.
2. For example, if region R2 is missing and the scores of the other anterior regions are known (for example L1 = 1, L2 = 0, R1 = 2) take the sum of the scores and divide it by the number of regions. Use the result for the missing region. In this case; (1 + 0 + 2)/3 = 1 = R2.
3. Please send the images to the steering committee.
4. Apply the correct ventilator strategy depending on the randomization and ARDS phenotype of the patient.

# Changes in statistical analysis compared to our previously published protocol.

In our previously published protocol, we described the use of Fleiss' kappa test as the statistical method for assessing our primary endpoint in this pilot phase. After publication, we determined that Cohen's kappa would be more appropriate, as it is better suited for assessing agreement between two raters (expert panel vs. local investigator) in mutually exclusive categories. Due to this change in the statistical test for our primary endpoint, we needed to revisit our sample size calculation. The initial sample size of 80 patients was determined to assess clinical feasibility and adherence to the study protocol, with a minimum of 20 patients in each treatment group. This calculation was based on the anticipated 1:1 ratio between 'focal' and 'non-focal' patients in both the control and personalized ventilation groups. To ensure we had sufficient power, we recalculated the sample size for our primary endpoint. With a slightly higher interobserver agreement among experts (κ = 0.9 vs. κ = 0.85), we determined that detecting a relevant reduction in κ to 0.7 between the expert panel and bedside clinicians would require a total of 60 patients to achieve 80% power at a one-sided α level of 0.05.

# Figure E1 – Total anterior LUS score in aligned and misaligned patients

| **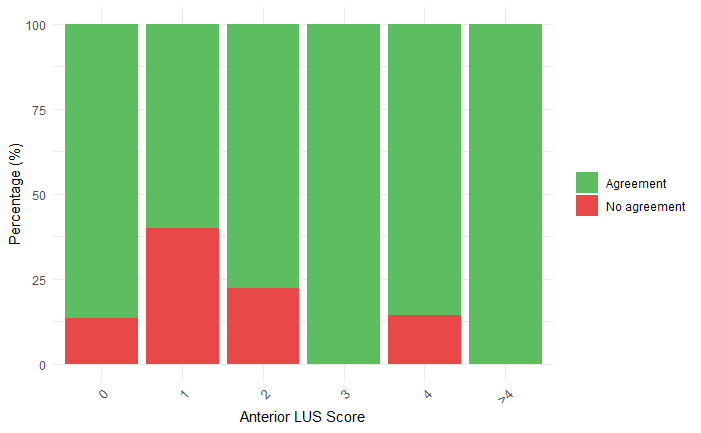** |
| --- |
| This figure displays the sum of the four anterior regions on the x-axis and the percentage of patients on the y-axis. Green indicates agreement between the treating physician and the expert panel, while red indicates no agreement. Most misclassifications are observed in patients with a total anterior LUS score of 1 or 2. *LUS = Lung Ultrasound.* |

# Figure E2 – Protocol adherence per patient.

| **Figure E2.1 – Non-focal patients randomized for personalized ventilation.** |
| --- |
| 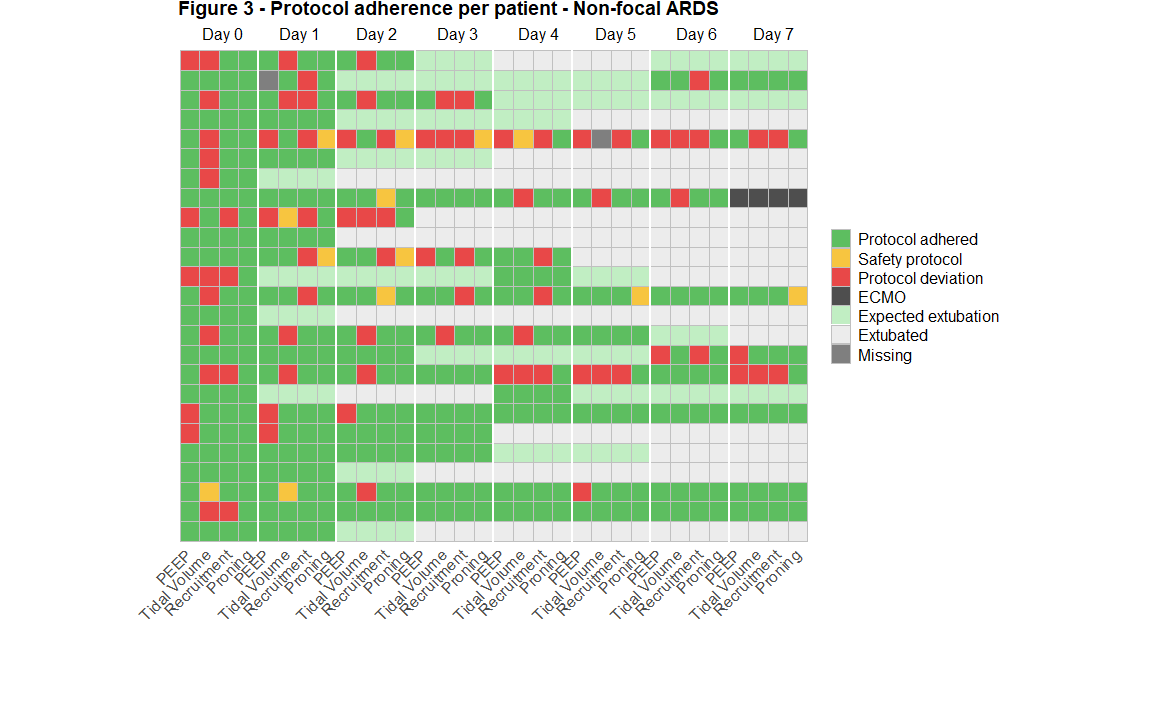 |
| **Figure E2.2 - Focal patients randomized for personalized ventilation.** |
| 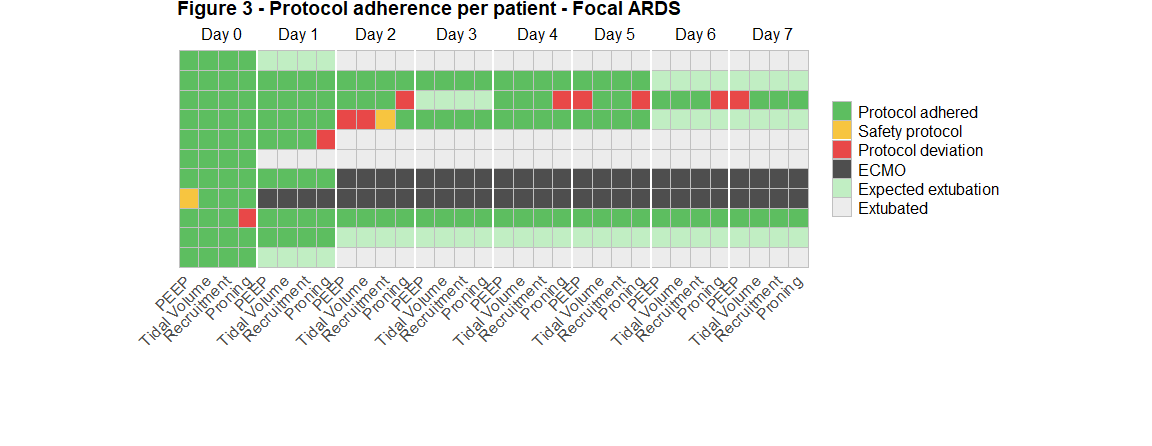 |
| **Figure E2.3 – Patients randomized for the control group.** |
| 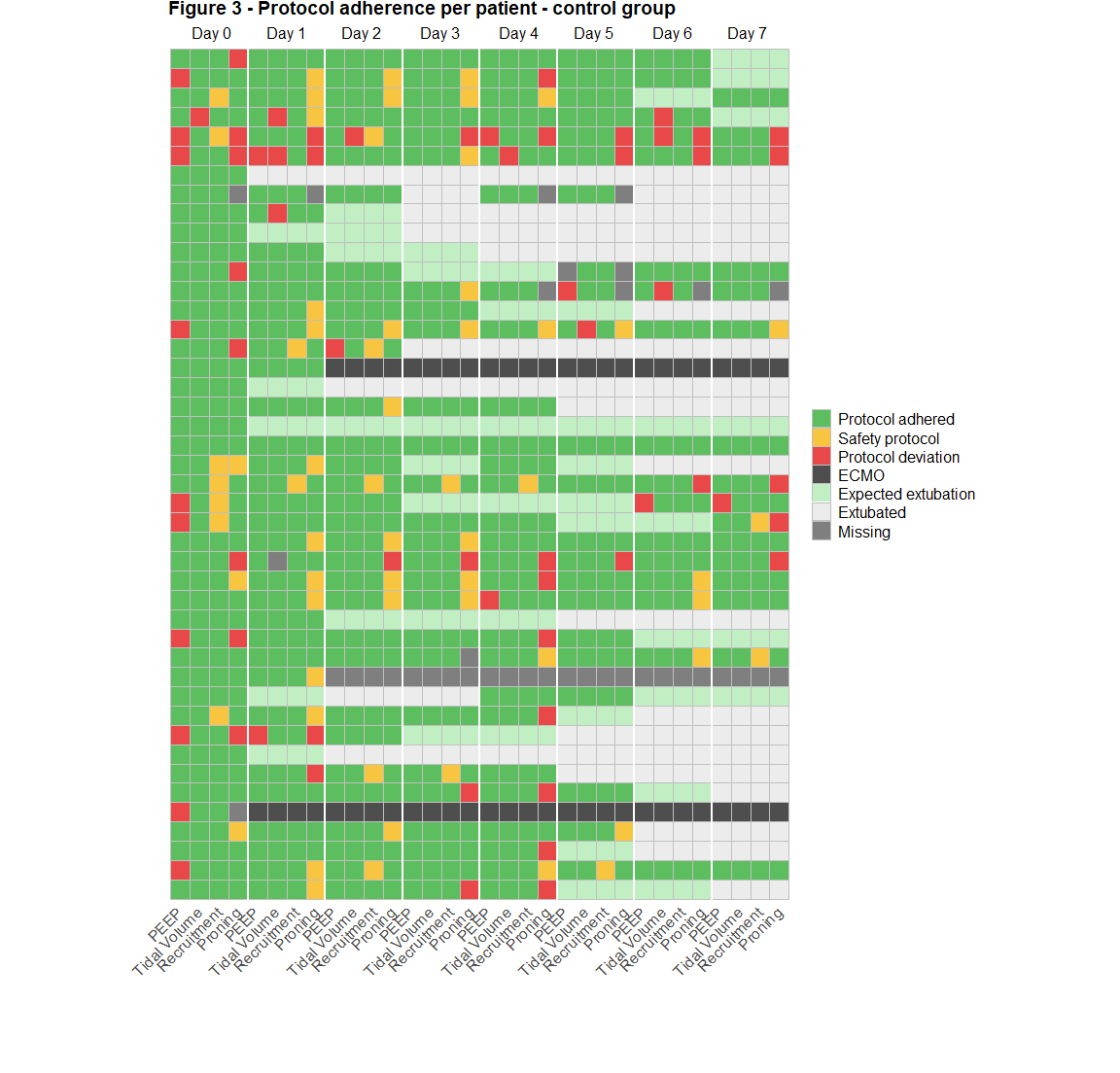 |
| Green indicates full adherence to the protocol, while orange denotes non-adherence due to safety concerns. Red represents non-adherence without safety concerns. Light green marks patients who were expected to be extubated within 48 hours based on ventilation parameters. Light grey indicates patients who were extubated, whether alive or deceased. Black signifies patients who were placed on ECMO, and dark grey represents missing data. *PEEP = positive end-expiratory pressure; ECMO = extracorporeal membrane oxygenation.* |

# Table E1 – Ventilation parameters per day, stratified for randomization arm, phenotype and mode of ventilation.

|  | **Personalized ventilation, focal** | | **Personalized ventilation, non-focal** | | **Standard of Care** | |
| --- | --- | --- | --- | --- | --- | --- |
| *Mode of Ventilation* | Mandatory | Supportive | Mandatory | Supportive | Mandatory | Supportive |
|  | **2 hours after randomisation** | | | | | |
| *PEEP (cm H2O)* | 8 [7-10] | 8 [6-8] | 15 [15-15] | 10 [10-10] | 10 [8-15] | 10 [10-13] |
| *Tidal volume (ml/kg pbw)* | 6.9  [6.7-7] | 6.8  [6.3-9.9] | 6.2  [5.6-6.7] | 7.1  [6.2-9.3] | 6.3  [6-6.8] | 6.7  [5.9-7.7] |
| *Patients with recruitment maneuvre (n)* | 0 (0%) | 0 (0%) | 14 (78%) | 0 (0%) | 6 (25%) | 1 (5%) |
| *Patients placed in prone position (n)* | 3 (75%) | 6 (86%) | 1 (6%) | 1 (14%) | 12 (50%) | 10 (50%) |
| *PaO2 / FiO2 ratio (mmHg)* | 92  [78-128] | 135  [115-171] | 159  [115-176] | 128  [111-170] | 139  [122-171] | 146  [106-188] |
|  | **Day 1** | | | | | |
| *PEEP (cm H2O)* | 8 [7-10] | 8 [6-8] | 15 [14-15] | 10 [10-10] | 10 [10-14] | 9 [8-12] |
| *Tidal volume (ml/kg pbw)* | 6.5  [5.5-7.1] | 9  [6.9-9.6] | 5.9  [5.7-6.4] | 6.8  [6.1-7.7] | 6.5  [5.7-7.2] | 7.7  [6.6-8.9] |
| *Patients with recruitment maneuvre (n)* | 0 (0%) | 0 (0%) | 10 (62%) | 0 (0%) | 1 (4%) | 1 (6%) |
| *Patients placed in prone position (n)* | 2 (50%) | 4 (67%) | 4 (25%) | 1 (11%) | 14 (56%) | 11 (61%) |
| *PaO2 / FiO2 ratio (mmHg)* | 175  [134-209] | 117  [116-120] | 183  [162-248] | 204  [168-281] | 175  [119-195] | 201  [143-240] |
|  | **Day 2** | | | | | |
| *PEEP (cm H2O)* | 10 [10-11] | 8 [8-8] | 15 [12-15] | 10 [8-12] | 12 [10-12] | 8 [6-10] |
| *Tidal volume (ml/kg pbw)* | 6.8  [6.3-7.3] | 5.4  [4.8-6.8] | 5.9  [5.7-6.5] | 6.2  [6-8] | 6.7  [6-7] | 7.2  [6.1-7.7] |
| *Patients with recruitment maneuvre (n)* | 1 (50%) | 0 (0%) | 8 (62%) | 2 (25%) | 4 (17%) | 1 (6%) |
| *Patients placed in prone position (n)* | 2 (100%) | 2 (40%) | 2 (15%) | 1 (12%) | 12 (52%) | NA (NA%) |
| *PaO2 / FiO2 ratio (mmHg)* | 137  [131-143] | 221  [150-233] | 195  [184-246] | 191  [148-247] | 195  [118-235] | 180  [171-208] |
|  | **Da**y **3** | | | | | |
| *PEEP (cm H2O)* | 8 [6-9] | 8 [8-8] | 15 [14-15] | 10 [6-10] | 10 [10-14] | 8 [6-10] |
| *Tidal volume (ml/kg pbw)* | 6.7  [2.7-6.9] | 7.3  [7.1-7.6] | 6.3  [6-6.7] | 7.5  [5.5-8.1] | 6.1  [5.8-7.1] | 6.5  [5.7-7.2] |
| *Patients with recruitment maneuvre (n)* | 0 (0%) | 0 (0%) | 2 (29%) | 0 (0%) | 1 (6%) | 1 (6%) |
| *Patients placed in prone position (n)* | 2 (40%) | 1 (50%) | 2 (29%) | 1 (9%) | 9 (53%) | 4 (22%) |
| *PaO2 / FiO2 ratio (mmHg)* | 195  [168-255] | 181  [152-209] | 191  [149-218] | 223  [181-250] | 161  [120-184] | 179  [165-208] |
|  | **Day 4** | | | | | |
| *PEEP (cm H2O)* | 8 [8-8] | 8 [8-10] | 15 [13-15] | 7 [5-8] | 10 [9-10] | 10 [8-12] |
| *Tidal volume (ml/kg pbw)* | 6.8  [6.8-6.8] | 6  [3.7-6.6] | 6.3  [6-6.8] | 7  [5.5-8.6] | 6.3  [6-7.3] | 7.3  [6.1-8.1] |
| *Patients with recruitment maneuvre (n)* | 0 (0%) | 0 (0%) | 2 (29%) | 0 (0%) | 1 (7%) | 0 (0%) |
| *Patients placed in prone position (n)* | 0 (0%) | 3 (50%) | 2 (29%) | 0 (0%) | 5 (33%) | 3 (14%) |
| *PaO2 / FiO2 ratio (mmHg)* | 184  [184-184] | 176  [135-215] | 142  [116-219] | 223  [174-261] | 167  [153-188] | 148  [139-221] |
|  | **Day 5** | | | | | |
| *PEEP (cm H2O)* | 8 [8-9] | 8 [6-9] | 14 [12-14] | 10 [6-11] | 10 [10-12] | 8 [8-10] |
| *Tidal volume (ml/kg pbw)* | 6.8  [5.6-7.1] | 5.5  [4.6-5.8] | 7  [6.7-7.3] | 6  [5.7-7.1] | 6.2  [5.9-7.1] | 7.4  [6.5-8.7] |
| *Patients with recruitment maneuvre (n)* | 0 (0%) | 0 (0%) | 1 (33%) | 0 (0%) | 1 (8%) | 0 (0%) |
| *Patients placed in prone position (n)* | 2 (50%) | 1 (33%) | 1 (33%) | 1 (9%) | 2 (17%) | 4 (20%) |
| *PaO2 / FiO2 ratio (mmHg)* | 177  [146-204] | 178  [163-209] | 118  [101-152] | 216  [168-247] | 164  [146-221] | 178  [153-238] |
|  | **Day 6** | | | | | |
| *PEEP (cm H2O pbw)* | 8 [7-8] | 8 [7-8] | 10 [10-13] | 10 [9-10] | 10 [10-12] | 8 [8-9] |
| *Tidal volume (ml/kg)* | 7.1  [4.5-7.5] | 5.2  [4.1-5.9] | 6.3  [5.9-6.3] | 7.3  [6.5-8.9] | 6  [5.5-7.7] | 6.9  [6.4-8] |
| *Patients with recruitment maneuvre (n)* | 0 (0%) | 0 (0%) | 1 (20%) | 0 (0%) | 0 (0%) | 0 (0%) |
| *Patients placed in prone position (n)* | 2 (67%) | 1 (25%) | 0 (0%) | 1 (12%) | 3 (27%) | 2 (13%) |
| *PaO2 / FiO2 ratio (mmHg)* | 182  [159-201] | 212  [178-225] | 180  [162-210] | 197  [135-251] | 159  [129-202] | 222  [196-243] |
|  | **Day 7** | | | | | |
| *PEEP (cm H2O)* | 8 [8-8] | 8 [7-8] | 14 [13-15] | 10 [9-10] | 10 [8-12] | 8 [6-10] |
| *Tidal volume (ml/kg pbw)* | 7.4  [4.9-7.4] | 6.4  [5.5-7.2] | 6.1  [5.8-6.7] | 7.2  [5.4-8.3] | 6.4  [6-6.8] | 7.2  [6.1-8.5] |
| *Patients with recruitment maneuvre (n)* | 0 (0%) | 0 (0%) | 2 (50%) | 0 (0%) | 2 (18%) | 0 (0%) |
| *Patients placed in prone position (n)* | 2 (67%) | 1 (25%) | 0 (0%) | 1 (12%) | 1 (9%) | 1 (8%) |
| *PaO2 / FiO2 ratio (mmHg)* | 175  [127-208] | 186  [176-195] | 126  [101-153] | 242  [151-278] | 150  [128-202] | 214  [193-230] |
| *PBW = predicted body weight, PEEP = positive end-expiratory pressure, PaO_2_ = Partial Pressure of Oxygen in arterial blood, FiO_2_ = fraction of inspired oxygen.* | | | | | | |

|  | |
| --- | --- |
|  | Cohen’s kappa |
| Local research team | 0.717 (95% CI: 0.53 – 0.9) |
| Expert 1 | 0.892 (95% CI: 0.77 – 1) |
| Expert 2 | 0.898 (95% CI: 0.79 – 1) |
| Expert 3 | 0.866 (95% CI: 0.74 – 0.99) |
|  | |

# Table E2 - Cohen’s kappa between expert opinion and local research team vs. expert panel (as golden standard).

# Table E3. Baseline characteristics of patients at inclusion stratified by subphenotype

|  | **Focal ARDS**  **n = 23** | **Non-focal ARDS**  **n = 57** |
| --- | --- | --- |
| **Baseline characteristics** |  |  |
| Age *(years)* | 55 (46-64) | 60 (49-75) |
| Female *(%)* | 7 (30%) | 26 (46%) |
| Duration of ARDS before randomization *(hours)* | 4 (2-8) | 4 (2-10) |
| Duration of ventilation before randomization *(days)* | 1 (1-2) | 1 (0-1) |
| Clinical frailty score | 2 (2-4) | 3 (2-5) |
| SOFA score | 9 (7-12) | 8 (7-11) |
| **Cause of ARDS (n (%))** |  |  |
| Pneumonia | 14 (61%) | 46 (81%) |
| Non pulmonary sepsis | 2 (9%) | 7 (12%) |
| Aspiration of gastric contents | 10 (43%) | 3 (5%) |
| Major trauma | 0 (0%) | 0 (0%) |
| Pulmonary contusion | 0 (0%) | 1 (2%) |
| Pancreatitis | 0 (0%) | 1 (2%) |
| Inhalation injury | 0 (0%) | 1 (2%) |
| Severe burns | 0 (0%) | 0 (0%) |
| Non cardiogenic shock | 0 (0%) | 0 (0%) |
| Drug overdose | 0 (0%) | 1 (2%) |
| TRALI | 1 (4%) | 1 (2%) |
| Pulmonary vasculitis | 0 (0%) | 0 (0%) |
| Drowning | 0 (0%) | 2 (4%) |
| **Ventilation data during LUS exam** | | |
| Supportive ventilation | 10 (43%) | 12 (21%) |
| PEEP *(cmH_2_O)* | 5 (5-8) | 8 (5-10) |
| Tidal volume *(mL/kg (PBW))* | 6.0 (5.0-7.0) | 6.0 (5.0-7.0) |
| PaO_2_ / F_I_O_2_ ratio *(mmHg)* | 115 (94-134) | 116 (91-154) |
| Driving pressure *(cmH_2_O)* | 13 (10-18) | 14 (9-20) |
| Global LUS score | 12 (8-13) | 20 (15-23) |
| The data is stratified by subphenotype defined by LUS at inclusion. Results are presented in median with IQR or number with percentages. *ARDS = Acute Respiratory Distress Syndrome;* *SOFA = Sequential Organ Failure Assessment; TRALI =* *Transfusion Related Acute Lung Injury; LUS = Lung Ultrasound; PEEP = positive end-expiratory pressure; PBW = predicted body weight; PaO2 = Partial Pressure of Oxygen in arterial blood; F_I_O_2_ = fraction of inspired oxygen.* | | |
